# A narrative review of the therapeutic and remedial prospects of cannabidiol with emphasis on neurological and neuropsychiatric disorders

**DOI:** 10.1186/s42238-024-00222-2

**Published:** 2024-03-18

**Authors:** Oluwadara Pelumi Omotayo, Yolandy Lemmer, Shayne Mason

**Affiliations:** 1 Human Metabolomics, Faculty of Natural and Agricultural Sciences, North-West University, Potchefstroom, South Africa; 2 Council for Scientific and Industrial Research (CSIR), Next Generation Health, Pretoria, South Africa; 3 Preclinical Drug Development Platform, Faculty of Health Sciences, North-West University, Potchefstroom, South Africa

**Keywords:** Anti-inflammatory cytokines, Cannabidiol (CBD), Neuroinflammation, Neurological infections, Neurological/neuropsychiatric disorders, Proinflammatory cytokines

## Abstract

**Background:**

The treatment of diverse diseases using plant-derived products is actively encouraged. In the past few years, cannabidiol (CBD) has emerged as a potent cannabis-derived drug capable of managing various debilitating neurological infections, diseases, and their associated complications. CBD has demonstrated anti-inflammatory and curative effects in neuropathological conditions, and it exhibits therapeutic, apoptotic, anxiolytic, and neuroprotective properties. However, more information on the reactions and ability of CBD to alleviate brain-related disorders and the neuroinflammation that accompanies them is needed.

**Main body:**

This narrative review deliberates on the therapeutic and remedial prospects of CBD with an emphasis on neurological and neuropsychiatric disorders. An extensive literature search followed several scoping searches on available online databases such as PubMed, Web of Science, and Scopus with the main keywords: CBD, pro-inflammatory cytokines, and cannabinoids. After a purposive screening of the retrieved papers, 170 (41%) of the articles (published in English) aligned with the objective of this study and retained for inclusion.

**Conclusion:**

CBD is an antagonist against pro-inflammatory cytokines and the cytokine storm associated with neurological infections/disorders. CBD regulates adenosine/oxidative stress and aids the downregulation of TNF-α, restoration of BDNF mRNA expression, and recovery of serotonin levels. Thus, CBD is involved in immune suppression and anti-inflammation. Understanding the metabolites associated with response to CBD is imperative to understand the phenotype. We propose that metabolomics will be the next scientific frontier that will reveal novel information on CBD’s therapeutic tendencies in neurological/neuropsychiatric disorders.

## Introduction

Plant-derived products, either alone or in combination with allopathic medicines, are increasingly being used to prevent and manage a wide range of ailments, including brain-related disorders. In recent years, cannabidiol (CBD), one of several cannabinoids obtained from *Cannabis sativa*, has been found to be of great medical importance. The alleged effectiveness of this chemical, along with other cannabinoids like delta-9-tetrahydrocannabinol (THC), in the treatment of several medical conditions has led to its rise in popularity. Moreover, CBD has been reported to be an antidote to certain disorders such as Parkinson’s disease, and seizures/epilepsy without exerting psychotropic effects, unlike THC, which is associated with psychoactive consequences (Fernández‐Ruiz et al. 2013; Porter et al. 2021).

Since the observations of Carlini et al. (1973) on the anticonvulsant effects of CBD, there has been a subsequent rise in the study of its properties, effects, and prospects, resulting in the growing appreciation of its therapeutic potential, as well as its anti-inflammatory and anti-oxidative properties (Aziz et al. 2022b; Cenova et al. 2022). According to Jean-Gilles et al. (2010), CBD has the ability to reduce the levels of pro-inflammatory cytokines, induce T-cell apoptosis, inhibit T-cell proliferation, and promote the migration and adhesion of immune cells. CBD also demonstrates the anti-oxidative potential of effecting redox balance through modification of the level and activity of oxidants and antioxidants, and captures free radicals or transforms them into inactive or less active forms (Atalay et al. 2019).

Moreover, the neuroprotective potential of CBD in various neurological disorders, such as ischaemic brain damage, has also been indicated (England and O'Sullivan 2021; Martínez-Orgado et al. 2021). CBD’s ability to inhibit inducible nitric oxide synthase (iNOS) protein expression and activation of nuclear factor kappa B (NF-_K_B) in the brain shows its neuroprotective tendencies (Bhunia et al. 2022; Ekiner et al. 2022). While extensive studies on the systemic therapeutic effects of CBD in the body have been done (Britch et al. 2021; Peng et al. 2022; White 2019), the potential of this phyto-cannabinoid on neurological/neuropsychiatric disorders requires further exploration regarding its effect on neuroinflammation that occurs as a consequence of these disorders. In addition to reviewing the therapeutic properties of CBD, and evaluating its potential as an anti-inflammatory agent, particularly in neurological/neuropsychiatric disorders, this article primarily identifies and states mechanisms through which CBD can help minimize inflammation caused by cytokine release. It also appraises the prospects of this phyto-cannabinoid in the management of brain-related disorders and discusses how the metabolomics approach (if adopted) can further CBD research.

### Cannabidiol

**Fig. 1:**
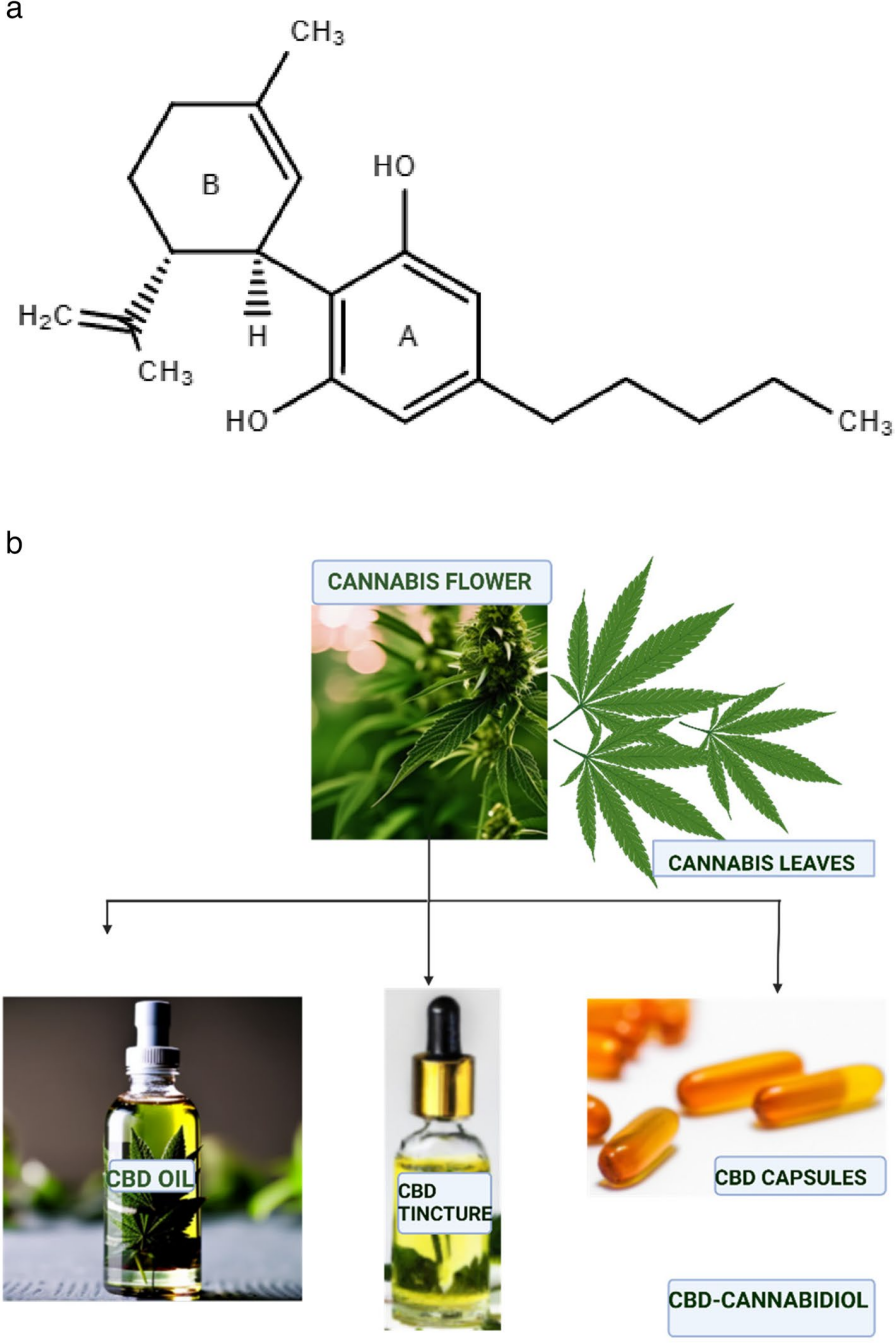
**a** Chemical structure of cannabidiol (CBD). **b** Forms of CBD derived from Cannabis

Cannabidiol (CBD), known scientifically as 2-[(1R,6R)-3-methyl-6-(prop-1-en-2-yl)cyclohex-2-en-1-yl]-5-pentylbenzene-1,3-diol, has the chemical formula of C_21_H_30_O_2_ and molecular weight of 314.5 g/mol. It is a terpenophenol and bicyclic compound consisting of a phenolic and cyclohexene ring, illustrated as A and B respectively in Fig. 1a. The pentyl chain and the location of the hydroxyl group in the phenolic ring (A), as well as the methyl group of the cyclohexene ring (B), significantly influence the chemical activity of the compound. For example, the hydroxyl groups enable it to bind to glutamine, threonine, and other amino acids through a hydrogen bond (ElSohly and Slade 2005; Jones et al. 1977; Mechoulam and Hanuš, 2002).

As an approved prescription drug for seizure disorders (Meissner and Cascella 2022), CBD is available in various forms such as oils and tinctures, capsules, and edibles (Fig. 1b). Clinically, it is often recommended for use in combination with antiepileptic therapy for the reduction of convulsive seizures in Dravet syndrome and treatment-resistant drop seizures in patients with Lennox–Gastaut syndrome (Devinsky et al. 2019; Privitera et al. 2021). While attention is commonly drawn to the natural product extracted from cannabis plants, another derivative of CBD, that is artificially/chemically synthesized – from biological processes using modified yeast or other ingredients such as limonene – is also used for various purposes ranging from cosmetic to medicinal (Abame et al. 2021; Stahl and Vasudevan 2020).

### Method of literature search

**Fig. 2:**
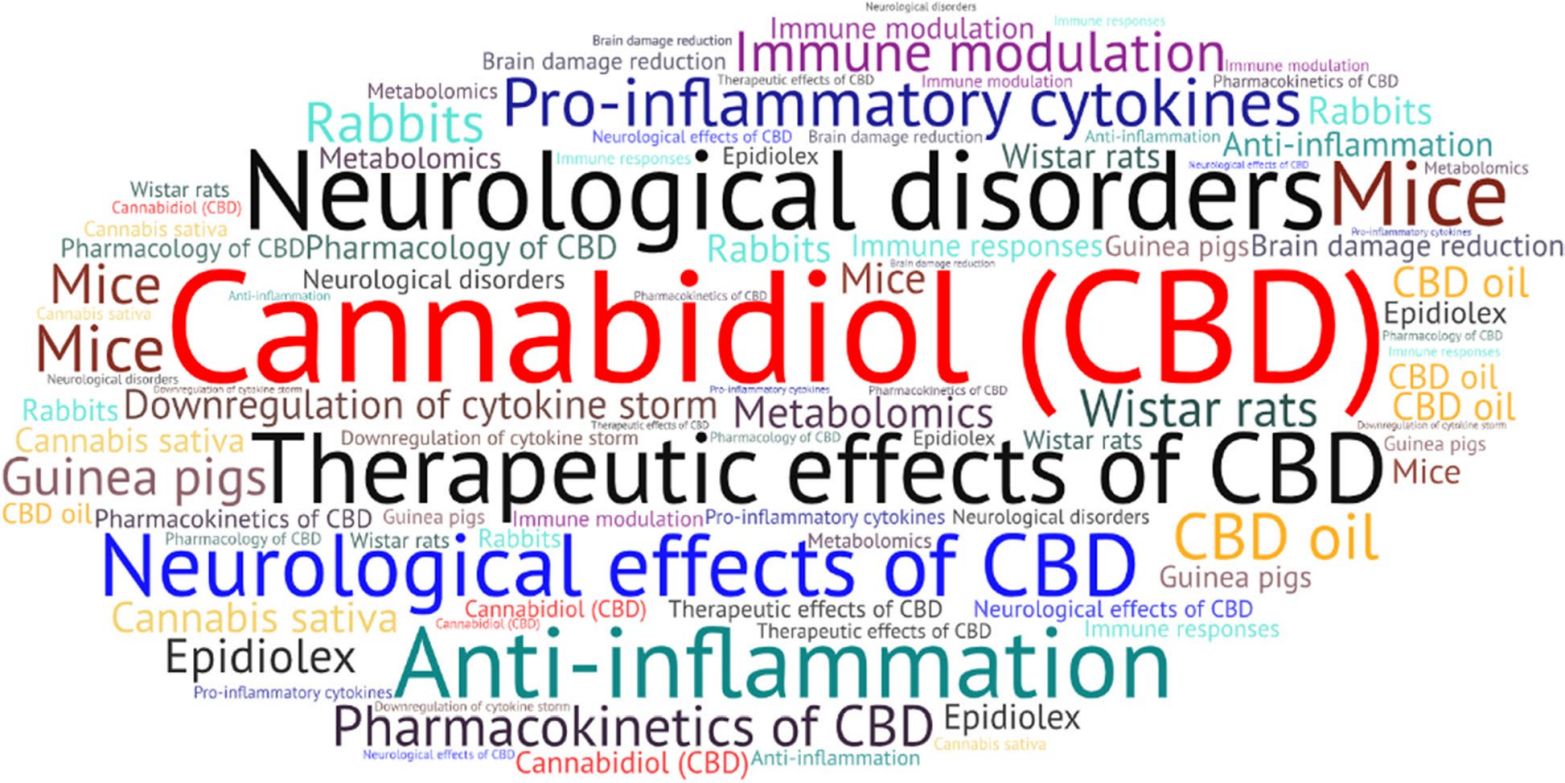
Word cloud showing the terms used in the included studies, the size of the words corresponds to the frequency of their use

This narrative review was conducted in accordance with the Preferred Reporting Items for Systematic Reviews and Meta-Analyses guidelines and reporting criteria (PRISMA). Following various scoping searches, a comprehensive literature search was conducted using available online scientific databases including Scopus, Science Direct, PubMed, Web of Science and Google Scholar. No time limit was used, and the keywords were: cannabidiol, epidiolex, cannabinoids, anti-inflammatory effects, neurological infections, neurological disorders, therapeutic effects of CBD, CBD effects on cytokines, and pro-inflammatory cytokines. Scientific papers, and commentaries written in English were also included. The initial search retrieved 414 articles, but after purposive screening and selection, only 170 of these (41%) which aligned with the objectives of this study were retained for inclusion. Respectively, Table 1 and Fig. 2 shows the screening and inclusion criteria (considered keywords) for this review.

**Table 1:** Target population, CBD-based therapeutic intervention, and search results

**Target population**	Human and laboratory animal models – mice, rats, rabbits, and guinea pigs
**CBD-based therapeutic intervention**	CBD, CBD oil, Epidiolex, *Cannabis sativa*
**Results**	Anti-inflammation, pro-inflammatory cytokines, downregulation of cytokine storm, immune modulation, immune responses, brain damage reduction, therapeutic effects of CBD, neurological effects, pharmacology, and pharmacokinetics of CBD

### Eligibility criteria

**Fig. 3:**
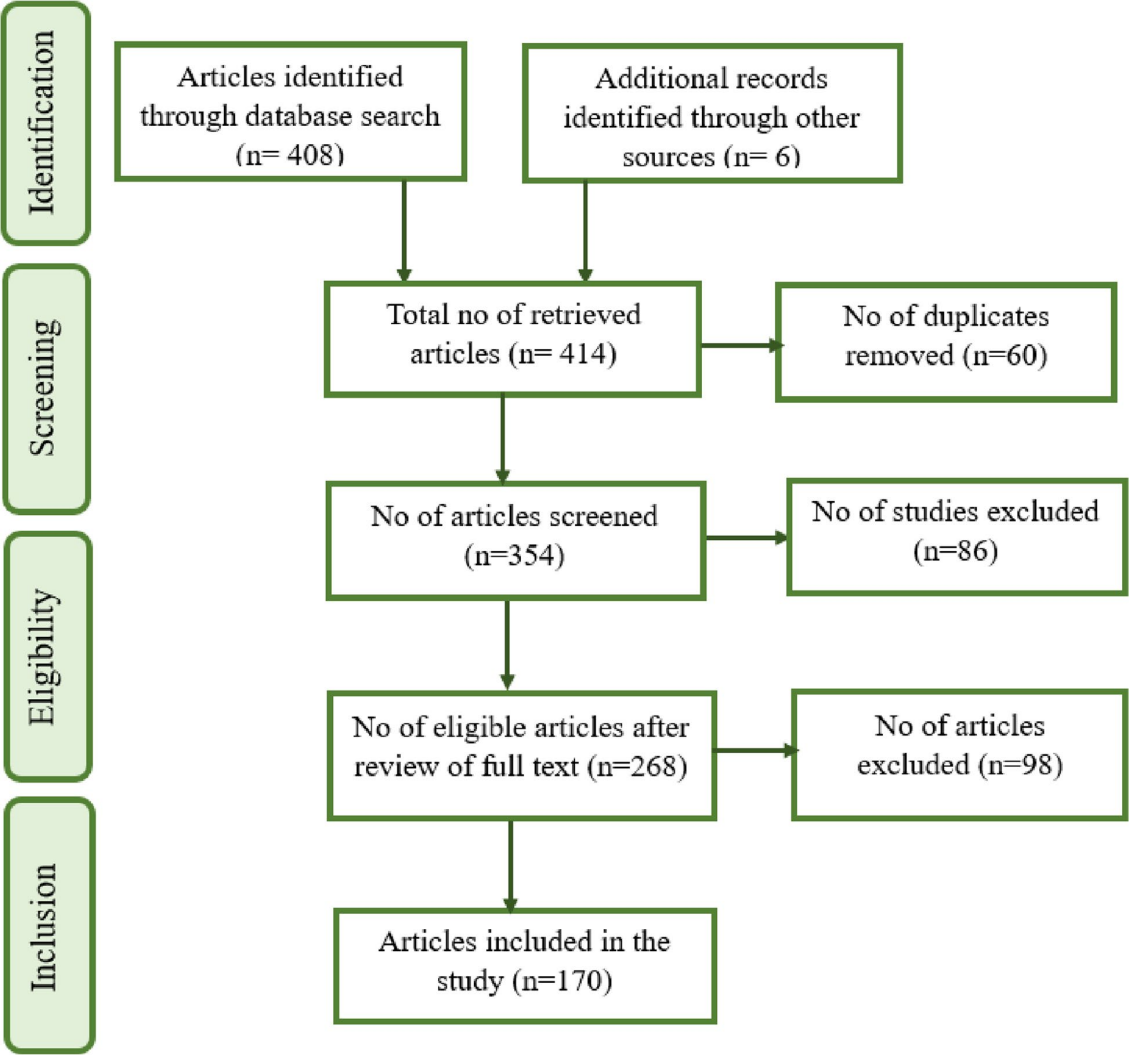
Flow diagram showing article selection process

Studies published in English language that were eligible and included in this review are those which considered the anti-inflammatory and therapeutic potentials of CBD, pharmacological/pharmacokinetic effects of CBD, effect of CBD on neurological disorders in human or animal model (s), or studies that assessed the impact of CBD on regulation of inflammation and cytokine release (Fig. 2). Articles that passed the initial screening were given full text review. Studies that were excluded were those that did not align with the aim of this study, did not deal with the effect of CBD, used standardised cannabis extracts containing both THC and CBD, or those with no appropriate controls to evaluate the influence of CBD alone. Figure 3 illustrates the article selection process.

### Data extraction

After the screening procedure, research articles that met the criteria were again reviewed and vital information was extracted including author(s), publication year, journals where the articles were published, experiments/aim of the study, target population (human or animal model), information pertaining to treatment used, interventions and dosage, route of administration and outcomes of the various included studies (Tables 2 and 3).

**Table 2:** Therapeutic properties of cannabidiol (CBD) in human cells, bacteria, and animal models

Properties	Experiments/aim	Dose/duration of treatment	Models/population	Results/outcomes	Author & year of publication
**Antimicrobial**	In vitro activity of CBD when used in combination with polymyxin B (PB) was examined against some Gram-negative bacteria (including *Klebsiella pneumoniae, Enterococcus faecium, Acinetobacter baumannii, Pseudomonas aeruginosa*) using the standard broth dilution method	4 µg/mL	Bacterial cells	The growth of these bacteria was inhibited with singular use of CBD but when used in combination with PB, a stronger antibiotic activity was observed	(Abichabki et al. 2021)
	CBD was used in combination with kanamycin against *Esherichia coli* and *Staphylococcus aureus*	1 or 5 µM	Bacterial cells	CBD functioned as an adjuvant agent in increasing antibiotic reaction against microorganisms	(Kosgodage et al. 2019)
	The antimicrobial activity of CBD against *Neisseria gonorrhoeae* and *S. aureus* was studied	35 µg	Bacterial cells	*Neisseria gonorrhoeae* was inhibited, and reduction was observed in the growth of *S. aureus*	(Blaskovich et al. 2021)
	The antibacterial properties of CBD against *Salmonella typhimurium* and *S. newington* was examined through bacterial kinetics, fluorescence microscopy, and biological assays	0.00125 – 1.25 µg/mL	Bacterial cells	CBD inhibited the growth of *S. typhimurium* and *S. Newington*	(Gildea et al. 2022)
	In vitro assay/microbial susceptibility test to study the effects of cannabinoids on *E. coli* and *S. aureus* was conducted	100 µL	Human cells: alveolar basal epithelial adenocarcinoma (A549), colon adenocarcinoma (Caco-2), hepatoblastoma cell line (Hep G2), epithelial breast adenocarcinoma cell line (MDA-MB-231) and hTERT-immortalized dermal fibroblast cell line (TelCOFS02MA, CRL4005)	Both organisms were susceptible to CBD effects with greater susceptibility seen in *S. aureus*	(Russo et al. 2021)
	In vitro/time-kill assay test to determine the effect of combined treatment of CBD and polymyxin against *Mycobacterium tuberculosis, S. lugdunensis, Micrococcus luteus, Enterococcus casseliflavus* and *Rhodococcus equi*	4 µg/mL	Bacterial cells	Combination of CBD and PB inhibited the organisms	(Abichabki et al. 2022)
	Time-kill assay to determine the potency of CBD against *Neisseria gonorrhoeae* and *Moraxella catarrhalis*	1 – 4 µg/mL	Bacterial cells	CBD displayed excellent activity against biofilms and showed significant antimicrobial activity against the two microorganisms	(Blaskovich et al. 2021)
**Anti-inflammatory**	Examination of the influence of CBD on the interactions of *Porphyromonas gingivalis, Filifactor alocis* and *Treponema denticola* with the immune system	1 µg/mL	Bacterial cells	CBD suppressed pro-inflammatory cytokines (IL-12 p40, IL-8, IL-6 and TNF) induced by *P. gingivalis* whereas it boosted the production of anti-inflammatory cytokines	(Gu et al. 2019)
	The effect of CBD on immune response markers associated with COVID-19 inflammation was examined in vitro	5 µg/mL	Human cells: alveolar epithelial cell line (A549), macrophage cell line (KG1)	Significant reduction in the production of IL-6 and IL-8	(Anil et al. 2021)
	CBD’s ability to interfere with interleukin (IL-12 and IL-10) production was studied in vivo and in vitro	15, 30 mg Kg^−1^ of CBD was administered intraperitoneally, and animals sacrificed after 1 h	Swiss male mice (18-20 g)	The production of IL-12 was enhanced by CBD, whereas it decreased IL-10 production	(Sacerdote et al. 2005)
	The anti-inflammatory effects of CBD and cannabigerol (CBG), singularly and in combination, were examined against lipopolysaccharide (LPS)-induced pulmonary inflammation	Intraperitoneal or oral administration at 10, 50/100 mg/kg for one day	Male adult Dunkin-Hartley guinea pigs (250-350 g)	CBD and CBG showed a clear anti-inflammatory effect in the lung by decreasing the potential of LPS in inducing neutrophil infiltration	(Cabrera et al. 2021)
	The effect of CBD on cell viability, intracellular calcium, and cytokine production was studied in rheumatoid arthritis synovial fibroblasts	≥ 5 µM	Rheumatoid arthritis synovial fibroblasts	CBD increased intracellular calcium, reduced cell viability and IL-6/MMP-3/IL-8 production of rheumatoid arthritis synovial fibroblasts	(Lowin et al. 2020)
	The reaction of CBD with th17 (characterized by production of interleukin-17) inflammatory autoimmune phenotype was examined	0.1 – 5 µM	MOG35-55 T cell line established from lymph node cells of C_57_BL/6 female mice	Significant decrease in the th17 phenotype, a decrease in the secretion of cytokines, including IL-17, whereas the secretion of the anti-inflammatory cytokine IL-10 was enhanced	(Kozela et al. 2013)
	The effects of CBD on the function of human sebaceous glands were explored	1 – 10 µM	Human sebocytes	Apart from suppressing sebocyte proliferation, CBD exerted anti-inflammatory effects such as inhibiting the expression of tumour necrosis factor (TNFα)	(Oláh et al. 2014)
**Antioxidative**	Animal model of mania was induced by D-amphetamine (D-AMPH) to investigate CBD effect on amphetamine-induced oxidative stress	Intraperitoneally for 14 days	Male Wistar rats (250-300 g)	CBD increased brain-derived neurotrophic factor and protected against D-AMPH-induced oxidative protein damage	(Valvassori et al. 2011)
	Consequence of CBD on oxidative stress, myocardial dysfunction, and interrelated signalling pathways was studied	Intraperitoneal administration of 1, 10/20 mg/kg CBD for 4–11 weeks	Male C57/BL6J Mice	Significant attenuation of oxidative stress observed	(Rajesh et al. 2010)
	In vivo evaluation was conducted on oxidative stress parameters in the brain and peripheral organs of male Wistar rats to study the effect of acute and extended administration of CBD	2.5, 5, and 10 mg/kg CBD was administered intraperitoneally once for the rats in the acute group and for 9 days for those in the extended group	Male Wistar rats (220-310 g)	Reduced oxidative stress in both the brain and peripheral organs	(Cassol-Jr et al. 2010)
	The antioxidant effect of CBD was explored in mice with carbon tetrachloride-induced liver fibrosis	4 and 8 mg/kg CBD was administered intraperitoneally twice a week for ten weeks	Male mice (C57BL/6 J)	Through adjustment of protein expression of gp9l and Nrf2, and suppression of the occurrence of lipid peroxidation, CBD exerted protective effect on CCl4-induced liver fibrosis in mice	(Run et al. 2022)
	The results of CBD administration in rats irradiated with UV were evaluated	0.6–62.3 mg of CBD was applied topically for 20 min every 12 h for four weeks	Male nude rats (Hsd: RH-Foxn1rnu, 260-302 g)	CBD penetrated the blood and caused a decrease in reactive oxygen species (ROS) generation as well as an increase in the activity of thioredoxin reductase and glutathione reductase	(Biernacki et al. 2021)
	The antioxidant effects of cannabidiol were examined in a rat model of ischaemic stroke	50, 100 and 200 ng of CBD was administered through intracerebroventricular injection for 5 days	Male Wistar rats (230-330 g)	At a dose of 100 ng/rat, CBD reduced the malondialdehyde level in rat brain as well as the infarction volume. It increased the activity of superoxide dismutase in the striatum and cortex	(Khaksar et al. 2022)
	The effect of CBD in a mouse model of cisplatin-induced nephropathy was investigated	Intraperitoneal administration of CBD at 2.5 – 10 mg/kg for 3 days	Mice	By chelating transition metal ions, which were involved in the Fenton reaction, to form extremely reactive hydroxyl radicals, CBD was observed to reduce ROS. It also minimized oxidative conditions by inhibiting the formation of superoxide radicals, which were greatly produced by xanthine oxidase (XO) and NADPH oxidase (NOX4 and NOX1)	(Pan et al. 2009)
**Neuroprotective**	Neuroprotective prospects of CBD were examined in the cortical neuron of rat cultures exposed to toxic levels of glutamate in vitro	2 – 4 µM	Female Wistar rat	CBD reduced glutamate toxicity	(Hampson et al. 1998)
	The potential of CBD to treat ethanol-induced neurodegeneration was conducted in vivo; neurodegeneration was assessed using fluoro-JadeB (FJB)	Transdermal gel application/intraperitoneal injection (20 mg/kg) twice daily for 3 days	Sprague Dawley rats (275-300 g)	FJB cells (neurodegeneration) reduced significantly after transdermal administration of 5% CBD	(Liput et al. 2013)
	The effect of CBD was examined in hypoxic-ischaemic newborn piglets; brain damage was studied by near-infrared spectroscopy	Singular and intraperitoneal administration of 0.1 mg/kg	Piglets	CBD administration after hypoxia–ischaemia reduced short-term brain damage	(Alvarez et al. 2008)
	A study was conducted to determine CBD effects on dopaminergic neurodegeneration of Parkinson’s disease using *Caenorhabditis elegans*	0.025, 0.05 and 0.1 mM	*C. elegans*	CBD enhanced lipid deposition thereby enhancing proteasome activity and reducing oxidative stress through the antioxidative pathway	(Muhammad et al. 2022)
	The neuroprotective effects of CBD were examined in rat model of Parkinson’s disease	3 mg/kg intraperitoneal administration for 2 weeks	Male Sprague–Dawley rats (≈250 g)	CBD provided neuroprotection against the degeneration of nigrostriatal dopaminergic neurons occurring in Parkinson’s disease	(García-Arencibia et al. 2007)
	The roles of CBD and CBG were examined in the regulation of hypothalamic neuromodulators	1000 nM	Rat hypothalamic hypo-E22 cells	CBD inhibited pro-opiomelanocortin, reduced the synthesis of hypothalamic norepinephrine, and reduced dopamine release	(di Giacomo et al. 2020)
	The therapeutic time window of the neuroprotective effects of CBD was tested in newborn hypoxic-ischaemic (HI) brain damage	1 mg/kg was administered subcutaneously for 7 days	C57BL6 Mice	CBD reduced ipsilateral hemisphere volume loss (IVHL), astrocyte damage, apoptotic damage. It also inhibited microglial population induced by HI	(Mohammed et al. 2017)
**Pro-apoptotic, anti-proliferative anti-migratory, and anti-invasive effects**	A study was conducted to determine the potency of CBD in reducing metastasis in vivo	1.5 µM	Human breast cancer cells (MDA-MB_231_)	CBD inhibited human breast cancer cell proliferation and invasion through differential modulation of the extracellular signal-regulated kinase, and reactive oxygen species pathways. It reduced primary tumour mass and lung metastatic foci	(McAllister et al. 2011)
	The impact of CBD on cancer cell invasion was examined using Matrigel invasion assays	10 µM	Human cells: HeLa, C33A and A549	CBD inhibited invasion of cancer cells via upregulation of tissue inhibitor of matrix metalloproteinases; invasion was reversed by antagonists to CB1 and CB2, and TRPV1	(Ramer et al. 2010)
	The consequences were investigated of the effect of CBD on neonatal iron overload on proteins, such as cytochrome c and Caspase 8, 9, and 3, which are involved in apoptotic pathways	10 mg/kg CBD administered intraperitoneally for 14 days consecutively	Pregnant Wistar rats	CBD reversed the increase in caspase 9,3 and cytochrome c caused by iron overload. It also displayed anti-apoptotic action	(da Silva et al. 2018)
	To investigate the ability of CBD in modifying the proapoptotic effects of UV irradiation in vitro	4 µM	Healthy and psoriatic human keratinocytes	Reduction in the apoptotic pathways, which was activated by UVB treatment. Inhibition of the expression of phosphorylated-p38 mitogen-activated protein kinase (p-p38), which promotes apoptosis	(Wójcik et al. 2021)

**Table 3:** Human clinical and animal model studies demonstrating the potential of CBD in neurological/neuropsychiatric disorders

Disorder	Study design	Oral dosage (mg/kg)	Outcomes	Author & year of publication
**Inflammation/Pain**	Randomized controlled trial of topical CBD for the treatment of thumb basal joint arthritis	6.2 mg/mL	Notable improvements in thumb basal joint arthritis-related pain	(Heineman et al. 2022)
	Double-blind randomized crossover trial of 24 patients with pain associated with a neurological diagnosis	2.5 – 120	Pain reduction	(Hunter et al. 2018)
	Double-blind, randomized, placebo-controlled single-patient cross-over trials to determine if CBD can relieve neurogenic symptoms	50 – 150	Significant decrease in pain	(Wade et al. 2003)
	Open-label study of 12 participants with severe somatoform and dysautonomia syndrome following human papillomavirus vaccination	150	Improved vitality and physical component scores	(Cuñetti et al. 2018)
	Examination of the efficacy of transdermal CBD for reduction of inflammation and pain in Sprague–Dawley rats	0.6 – 62.3	Reduced inflammation and pain-related behaviours	(Hammell et al. 2016)
**Post-traumatic stress disorder (PTSD)**	An open-label trial was conducted on 11 patients with PTSD	22 – 28 oral capsules or 1.5 spray	Reduced PTSD symptoms in 10 patients	(Elms et al. 2019)
	The effect of CBD on PTSD-like behaviours in mice was investigated in adult male C57BL/6 J mice	10	CBD produced anti-PTSD-like actions, attenuated trauma-related fear memory and anxiety-like behaviour	(Han et al. 2022)
**Schizophrenia**	A double-blind study of patients with schizophrenia treated with CBD and amisulpride	200	Reduced symptoms with less weight gain and minimal increase in prolactin	(Leweke et al. 2012)
	A double-blind study of patients with schizophrenia treated with CBD in conjunction with the psychotic treatment	1000	Decreased psychotic symptoms	(McGuire et al. 2018)
	Administration of CBD during peri-adolescence stage to determine its ability to prevent schizophrenia-like behavioural activities in an animal model of schizophrenia (Wistar rats and spontaneously hypertensive rat-SHR)	0.5 – 10	CBD prevented SHR’s hyperlocomotor activity and cognitive schizophrenia symptoms	(Peres et al. 2018)
**Substance Abuse and withdrawal**	Double-blind placebo-controlled study in which Epidiolex was administered to abstinent heroin users	400/800	Reduced cue-induced heroin craving and anxiety	(Hurd et al. 2019)
	Double-blind placebo-controlled study: inhaler containing CBD was administered to cigarette smokers	0.4	Reduction in the number of cigarettes consumed after 7 days	(Morgan et al. 2013)
	A double-blind crossover study in which 30 cigarette-smoking participants were given oral CBD capsules	800	Reduced pleasantness of cigarettes	(Hurd et al. 2019)
	An open-label study including 8 cannabis users	600 – 1200	Cannabis abstinence was observed in 4 out of 8 patients	(Pokorski et al. 2017)
**Insomnia**	Large retrospective case series: CBD was applied clinically as an adjunctive treatment to 25 adult patients	25 – 75	Sleep scores improved within the first month of administration	(Shannon et al. 2019)
	Case report: CBD oil was singularly administered to a 10-year-old female patient	25	Symptoms of insomnia were suppressed during administration	(Shannon and Opila-Lehman 2016)
	Case report: administration of CBD oil adjunctively to a 20-year-old patient with insomnia for a duration of six months	200 – 800	Increased duration of sleep	(Berger et al. 2020)
	CBD was administered to male Wistar rats to determine its effects on sleep	10 µg	CBD administered during lights-on period caused a decrease in rapid eye movement sleep and an increase in wakefulness. No sleep changes were observed in the dark period	(Murillo-Rodríguez et al. 2006)
**Anxiety**	Randomized, crossover, double-blind, placebo-controlled study: CBD was administered to 10 patients with social anxiety disorder	400	Suppressed anxiety in patients	(Crippa et al. 2011)
	Double-blind placebo-controlled study: CBD was tested for anxiolytic properties in 40 participants	300	Attenuated anxiety	(Zuardi et al. 1993)
	Randomized double-blind placebo-controlled trial: To investigate the anxiolytic effects of CBD in 60 participants	100, 300 and 900	Reduced anxiety in patients treated with 300 mg CBD but the reverse was the case with 100 mg and 900 mg	(Linares et al. 2018)
	A large case series: CBD administered to 72 patients with anxiety concerns	27 – 75	Anxiety scores decreased from the first month through the course of treatment in 57 patients	(Shannon et al. 2019)
	The behavioural effects of CBD were investigated in male *Fmr1* KO mice	5 or 20	Reduced anxiety-like behaviour in mice	(Zieba et al. 2019)
**Seizures/Epilepsy**	Open-label interventional trial: CBD was administered to 214 patients (1–30 years) with severe, intractable, childhood-onset, treatment-resistant epilepsy Double-blind, placebo-controlled trial: CBD administered to 120 children and young adults with Dravet syndrome	2 – 50 20	Reduction in the frequency of seizures. Reduction in the frequency of convulsive seizures	(Devinsky et al. 2016) (Devinsky et al. 2017)
	Prospective multi-centre open-label study on 55 adults and children (1–30 years) with Dravet syndrome	5 – 50	Monthly convulsive seizure decreased by 59.1% at week 48	(Devinsky et al. 2018)
	A prospective open-label study of 40 patients (1–17 years) with drug-resistant epilepsy	5 – 25	Significant improvement was reported in 12 patients while others developed either serious complications or withdrew from the study	(Chen et al. 2018)
	A prospective multi-centre open-label study involving 607 participants (average of 13 years) with drug-resistant epilepsy	2 – 50	Reduction in the frequency of convulsive seizures	(Szaflarski et al. 2018)
	Retrospective study of 210 patients with epilepsy	2.9 – 5.8	Reduced frequency of convulsive seizures	(Porcari et al. 2018)
	The anti-seizure effect of CBD was investigated in mice and rat model of pilocarpine-induced status epilepticus	10	CBD attenuated maximum seizure severity	(Patra et al. 2019)
	The effect of cannabidiol on seizure was investigated in a mouse model of Dravet syndrome	100 – 200	Reduced seizures and autistic-like social deficits	(Kaplan et al. 2017)
**Parkinson’s disease (PD)**	Case report: The effects of CBD in patients receiving treatments for PD and psychosis was examined	75 – 300	Quality of life was improved, especially in the group given 300 mg/kg oral CBD	(Chagas et al. 2014)
	The consequences of chronic CBD treatment were examined on neurodegenerative and neuroinflammatory processes, and motor deficits in a classic toxic model of PD	10	Reduction of nigrostriatal degeneration, improvement of motor performance, and damping of the neuroinflammatory response	(Giuliano et al. 2021)
	A case series involving four PD patients with rapid eye movement sleep behaviour disorder (RBD)	150 – 300	Significant reduction in the frequency of RBD	(Rieder 2020)
	Double-blind placebo-controlled clinical trial of the efficacy and safety of CBD for RBD in PD	75 – 300	Substantial improvement in sleep satisfaction	(de Almeida et al. 2021)
	Effects of chronic CBD treatment was evaluated on PD-associated neurodegenerative and neuroinflammatory processes	10	CBD enhanced the endogenous neuroprotective response of ciliary neurotrophic factor	(Giuliano 2021)
	The effect of CBD on PD in a transgenic mouse model was investigated		CBD protected the substantia nigra and significantly improved motor deficits of PD model	(Zhao et al. 2022)
**Huntington’s disease**	Double-blind, randomized cross-over design: a controlled clinical trial of CBD in Huntington’s disease	10	No significant effect nor toxic effects were observed	(Consroe et al. 1991)
	The neuroprotective effects of cannabinoids including CBD was examined in male Sprague–Dawley rats	5	CBD prevented 3NP-induced striatal damage (relevant for Huntington’s disease)	(Sagredo et al. 2007)
**Depression/Mood disorder**	An observational study to assess the effect of CBD users with anxiety and/or depression	0.8	Reduction in self-reported depression as well as improved quality of life was reported	(Martin et al. 2021)
	The antidepressant-like effects of CBD were examined in male Swiss mice	3 – 100	At 30 mg/kg, CBD reduced immobility time in forced swimming test conducted	(Zanelati et al. 2010)
	The anti-depressant effect of CBD was investigated in rat model of depression (Wistar-Kyoto rat)	15 – 45	CBD caused an increased locomotion showing improvement in low motivation of the rats to explore	(Shoval et al. 2016)
**Cognitive impairment**	Randomised double-blind, parallel- group and placebo-controlled clinical trial to determine the effect of CBD on cognitive function	400 and 800	CBD had no significant cognitive effect	(Lees et al. 2023)
	Randomised, double-blind, placebo-controlled trial to evaluate the efficacy of CBD on cognitive deficits	800	No significant evidence	(Rizkallah et al. 2022)
	Randomised, double-blind, placebo-controlled trial to determine the effect of CBD on cognitive impairment and THC-elicited psychosis	600	CBD preserved hippocampal-dependent memory and decreased THC-elicited psychotic symptoms	(Englund et al. 2013)
	The memory-rescuing effects of cannabidiol was examined in memory-impaired male Wistar rats	5 or 10	Memory recovery and improved recognition memory	(Fagherazzi et al. 2012)
	The potential of CBD in the prevention of hippocampal neurodegeneration and cognitive deficits caused by brain ischemia in male Swiss albino mice was investigated	3, 10 and 30	CBD prevented neuronal death caused by ischemia	(Schiavon et al. 2014)
**Dementia/Alzheimer’s disease**	Double-blind placebo-controlled clinical trial to determine the efficacy of CBD in the treatment of early-stage dementia when administered daily over a period of 12 weeks	200 – 300	Improved quality of life, prognoses, and treatment outcomes	(Bartschi et al. 2023)
	A Placebo controlled randomized double-blind clinical trial to study the effects of CBD oil on behavioural disturbances in patients suffering from dementia	295	Improvements in patients, reduction in sleep disturbances, reduced agitation, and aggression	(Hermush et al. 2022)
	The effects of long-term administration of CBD on learning and anxiety was examined in female Alzheimer’s disease mouse model	20	Spatial learning enhancing effect was caused by CBD	(Chesworth et al. 2022)

This study addresses the pharmacology, modes of action, absorption, metabolism, tolerance, and therapeutic qualities of cannabidiol (CBD). In addition, this phytocannabinoid's prospects and potentials in neurological disorders were examined, and the potential of CBD in treating neurological disorders was evaluated.

### Pharmacology and mechanisms of action of CBD

Being a drug with many targets, CBD interacts with several signalling systems and modulates the γ-aminobutyric acid type A receptors, and 5-hydroxy tryptophan 1A receptors (5-HT_1a_), which are involved in neurotransmission and neuromodulation, respectively (Ghit et al. 2021; Hilaire et al. 2010; Russo et al. 2005). However, it has been observed that CBD inhibits endocannabinoid signalling (Straiker et al. 2018). The endocannabinoid system regulates many physiological activities in the human body, and it involves the endogenous cannabinoids (endocannabinoids), cannabinoid receptors, and enzymes as its main components (Lu and Mackie 2016). The endocannabinoids have been described as molecules produced by the body which are similar to cannabinoids. The two main endocannabinoids identified so far are anandamide, also known as N-arachidonoylethanolamine (AEA), and 2-arachidonoyl glycerol (2-AG), which are produced as needed and help to keep internal functions running smoothly (Rodríguez de Fonseca et al. 2005). The CB1 receptors (found mostly in the central nervous system) and the CB2 receptors (found mostly in the immune cells- peripheral nervous system) receives the endocannabinoids and signal to the endocannabinoid system to act while the fatty acid amide hydrolase (FAAH) and monoacylglycerol acid lipase (MGAL) breaks down the endocannabinoids after carrying out their function (the FAAH breaks down AEA while the MGAL breaks down 2-AG) (Basavarajappa 2007).

CBD acts as an antagonist of cannabinoid receptor 1 (CB1) and cannabinoid receptor 2 (CB2). CB1 receptors are highly expressed in the hippocampus and modulate seizure activity, whereas CB2 receptors are localized in immune cells. This implies that CBD modulates neurotransmission, immune/inflammatory pathways, and other peripheral functions; thereby protecting against cellular damage that occurs during immune responses (Peyravian et al. 2020).

It has been established that CBD acts as a negative allosteric modulator of the CB1 receptor, and this is achieved by modifying the activity of a receptor on a distinct site from the antagonist binding site/agonists (Laprairie et al. 2015). It is important to note that allosteric modulators of CB1 receptors are capable of treating disorders of the peripheral and central nervous system while preventing the negative effects linked with antagonism of these receptors (Laprairie et al. 2015). At higher concentrations, CBD acts as an inverse agonist of G protein-coupled receptors (GPR, GPR6, GPR12, and 5-HT_1a_), which are phylogenetically related to CB receptors, and are novel therapeutic targets for CBD (Laun et al. 2019). CBD also binds to transient receptor potential vanilloid (TRPV1), impeding synaptosomal uptake of noradrenaline, serotonin, and dopamine (Bisogno et al. 2001; De Petrocellis et al. 2011), blocks low-voltage-activated (T-type) Ca^2+^ channels, acts on mitochondria Ca^2+^ stores and constrains the activity of FAAH and it also stimulates the action of the inhibitory glycine receptor (Massi et al. 2008).

### Absorption, metabolism, and tolerability of CBD

Through inhalation, CBD is rapidly absorbed with a higher bioavailability by an average of about 35% than oral bioavailability – reported to be low (~ 6% in humans) (Davidson 2022; Lucas et al. 2018). Thus, when ingested, CBD has a lower absorption rate than when inhaled, due to first-pass hepatic metabolism (Della Rocca et al. 2022). In addition to the frequently used oral and intranasal routes, CBD can also be administered through the sublingual and rectal routes. However, the potency of these routes still needs to be established as few studies and have described the pharmacokinetics of CBD administered rectally.

When administered transdermally, significant accumulation of CBD was observed in the skin as well as in the underlying muscle; steady-state levels were reached at about 24 h and lasted for 72 h (Lodzki et al. 2003). In their study, Hosseini et al. (2021) observed that administration of CBD as a sublingual wafer (50 mg CBD) to healthy human volunteers resulted in maximum concentrations of the drug after 4 h with an estimated terminal elimination half-life of 6 h. More studies are still required on the delivery of CBD through injections; however, Fu et al. (2022) found that intramuscular injection of CBD nanocrystals resulted in a 2.0- to 2.2-fold greater concentration of CBD in fasted male Sprague–Dawley rats within 24 h, compared to oral administration.

With a biological half-life of about 18–32 h, up to 1500 mg of CBD per day can be well tolerated in the body without adverse effects, especially on the brain (Machado Bergamaschi et al. 2011; Welty et al. 2014). Through the renal and biliary systems, CBD is metabolized greatly in the liver by the isoenzymes UGT1A7, UGT1A9, UGT2B7 and cytochrome P450 enzymes (CYP2B6, CYP2C19, CYP2D6, CYP2J2, and CYP3A4), thus forming several metabolites such as the 6α- and 6β-hydroxy isomers and 7-hydroxy cannabidiol (Chayasirisobhon 2020; Jiang et al. 2011; Welty et al. 2014).

Psychotic adverse effects of CBD in humans have not been reported as it is noted to be well tolerated (Machado Bergamaschi et al. 2011). In a study to evaluate the pharmacokinetics and safety of CBD oral solution in paediatric patients with treatment-resistant epilepsy – defined as recurrent seizures despite adequate trials of three or more antiepileptic drugs and one or more prior adequate treatment courses with two or more antiepileptic drugs in combination, Wheless et al. (2019) confirmed the tolerability of CBD, as they observed how CBD was well metabolized in more than sixty human patients (age 1–17) included in the study, with few adverse effects such as somnolence, diarrhoea, and anaemia.

When used as an adjunctive treatment with anti-seizure medications, as evidenced in the study of Devinsky et al. (2018), CBD reduced the frequency of seizures in four epilepsy etiologies (Doose syndrome, Aicardi, Dup 5q and CDKL 5 deficiency disorder), with moderate side effects such as diarrhoea, fatigue, and somnolence. Similarly, the study of Kochen et al. (2023) confirms the safety and effectiveness of cannabidiol as an adjuvant in adult patients with drug-resistant focal epilepsy with adverse effects including diarrhoea, somnolence, and decreased appetite (Kochen et al. 2023). It is therefore important to emphasize that despite the effectiveness and safety of CBD, minor side effects such as those stated may occur.

### Therapeutic properties of CBD

The application of CBD, singularly and in combination with other medicines in the management of various health disorders, has shown beneficial effects including antibacterial, anti-inflammatory, anti-oxidative, neuroprotective, proapoptotic, anti-proliferative, anti-migratory, and anti-invasive capabilities (Aziz et al. 2022a). That is, CBD can act against specific pathogens, block pro-inflammatory cytokines, capture and convert free radicals into passive forms, safeguard against specific neurological conditions, and prevent the invasion and migration of cancer cells (Aziz et al. 2022a; Cabrera et al. 2021).

In neuroinflammation, activated microglia can migrate toward a lesion (affected neuron) and secrete inflammatory mediators, neurotrophic factors, and reactive oxygen species (ROS) which can exert beneficial or detrimental effects on the surrounding cells (Carbonell et al. 2005; Stence et al. 2001). The activation of microglia has been linked to increased inflammatory response and could result in progressive neuron damage. Hence, persistent and overactivation of microglia has been linked to multiple neurodegenerative diseases (Kim and Joh 2006).

However, CBD has been reported to be a good modulator of microglia cell migration in inflammation. According to Martín-Moreno et al. (2011) who studied and compared the effects of CBD with those of other cannabinoids on microglial cells in Alzheimer’s disease, CBD was able to regulate microglial cell function in vitro while it also caused some beneficial effects in an in vivo model of Alzheimer’s disease. Victor et al. (2022) examined the effect of CBD on microglial inflammation activation and neurogenic response in seizures and in the absence of seizures it was reported that CBD reduced microglial migration and accumulation to the hippocampus.

Table 2 lists various studies performed on human cells, bacteria and animal models that have revealed some of the therapeutic characteristics of CBD. Table 3 describes human clinical trials and studies that were carried out on animal models to assess the potential of CBD for managing various health issues, including brain-related disorders.

### Prospects of CBD in neurological disorders

In neurological disorders, an immune response-based defense is generated, which induces systemic inflammation by the continuous production of pro-inflammatory cytokines and granulocyte–macrophage colony-stimulating factor, thereby resulting in changes in cells/tissue and the possibility of organ damage (Misri et al. 2022; Seltzer et al. 2020). Signalling on the immune system involves various pathways that achieve the release of chemokines and cytokines through mitogen-activated protein kinase (MAPK), Janus kinase-signal transducer and activator of transcription (JAK-STAT), and NF-_K_B (Hu et al. 2021).

Owing to its action as an inverse agonist of the CB2 receptor (Thomas et al. 2007), CBD can help minimize inflammation caused by cytokine release through the following mechanisms:

- Reduction of oxidative stress
- Regulation of the cerebral adenosine
- Downregulation of tumour necrosis factor (TNF-α) receptor
- Restoration of brain-derived neurotrophic factor (BDNF)
- Restoration of 5-hydroxytryptamine (5-HT) levels in the brain.

**Fig. 4:**
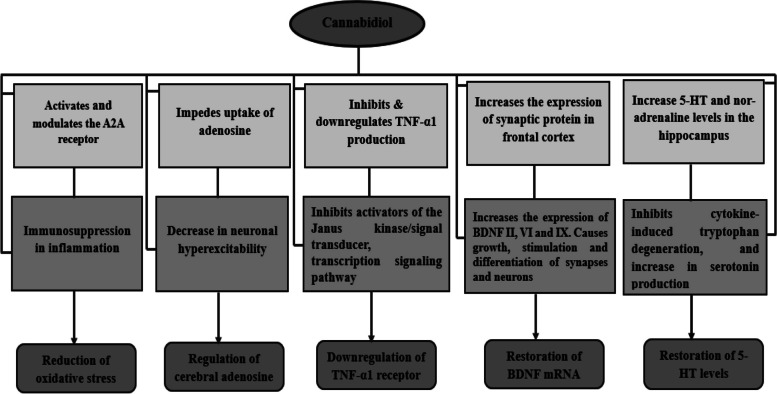
Cannabidiol’s remedial mechanism of action in neurological disorders

These five mechanisms are discussed below; Fig. 4 summarises CBD’s remedial activity in brain-related disorders in relation to these mechanisms.

### Reduction of oxidative stress and inflammation

In order to mitigate the effect of pathogens and start tissue repair, excessive production of ROS by the immune system may occur, thus leading to oxidative stress (Juan et al. 2021). In brain-related disorders, the cell and tissue damage, inflammation, and decreased performance in cognitive function which results from increased ROS production and oxidative stress, leads to brain impairment over time, by initiating cell death and disrupting the blood–brain barrier (Salimi and Klein 2019).

However, CBD can activate the adenosine A_2A_ receptors (A_2A_), which are distributed in the brain-striatum, nucleus accumbens, and olfactory tubercule, that could lead to anti-inflammatory activity and reduce oxidative stress (Hao et al. 2015; Magen et al. 2009; Vallée et al. 2017). A_2A_ takes part in several pathological reactions, exhibits anti-inflammatory characteristics, and modulates inflammatory processes, thus the activation of the adenosine receptor is a major mechanism of immunosuppression in inflammation.

In the in vivo studies of Hao et al. (2015) in which the effect of CBD was examined in a mouse model of Doxorubicin-induced cardiomyopathy and that of Vallee et al. (Vallée et al. 2017) who examined the interactions of CBD and peroxisome proliferator activated receptor γ (PPARℽ) on oxidative stress and neuroinflammation, it was found that CBD is capable of reducing oxidative stress by decreasing mitochondrial dysregulation and reducing the expression of ROS-generating nicotinamide adenine dinucleotide phosphate (NADPH) oxidase isoforms. This indicates that the reduction of mitochondrial dysfunction (which causes detrimental effects on the cells and plays a major role in the severity of several pathological conditions such as neurogenerative diseases) and the decrease in the expression of NADPH results in modulation of ROS production thereby regulating and reducing inflammation.

Vallée et al. (2017) also emphasized the suppression of pro-inflammatory cytokines by the activation of peroxisome proliferator-activated receptors which regulates inflammation. In the in vivo study of Pan et al. 2009, that was targeted at investigating the effects of CBD in a mouse model of Cisplatin-induced neuropathy (in which Cisplatin caused an increase in the generation of ROS, expression of NOX4 and NOX1, apoptosis, formation of nitrotyrosine, tumour necrosis factor α and interleukin-1 β in the kidneys of mice and impaired renal function), it was observed that CBD caused a decrease in oxidative stress and inflammation, thus resulting in the attenuation of Cisplatin-induced nephrotoxicity and improved renal function.

### Regulation of the cerebral adenosine system

Adenosine is found in the extracellular fluid of most bodily tissues, including the brain, and reacts to pathogen invasion, stress, and damage. It occurs in normal cells in concentrations ranging from 1 to 10 mmol/L (Zimmerman et al. 2011); however, these concentrations rise quickly in response to cellular damage, and an excessive/chronic overproduction of adenosine disrupts the adenosine system, resulting in immune imbalances and immunosuppression. At high concentrations, adenosine becomes hazardous and toxic to the immune system, resulting in dysfunction and autoimmune disorders which adversely affect the brain (Borea et al. 2017).

Adequate concentrations of adenosine in the brain have significant effects on its health and development, and a person’s mood; hence, an adenosine imbalance may lead to detrimental effects on brain tissues (Figler et al. 2011; Zhang et al. 2011; Zhang and Xia 2012). According to Borea et al. (2017), as well as Feoktistov and Biaggioni (2011), excess production of adenosine has been associated with certain neurodegenerative disorders such as Parkinson’s disease. This is because excessive adenosine generation causes overexpression of the A2B receptors, thereby increasing inflammation because A2B most often plays an opposite role to that of A2A. In high concentrations, adenosine initiates the production of inflammatory cytokines such as transforming growth factor beta (TGFβ), tumour necrosis factor alpha (TNF-α), and interferon-gamma (IFN-γ) (Sauer et al. 2017).

CBD is able to regulate the cerebral adenosine system by impeding the uptake of adenosine through obstruction of the equilibrative nucleoside transporter ENT1 (Pandolfo et al. 2011). ENT1 is linked with the synaptic uptake of adenosine; thus, inhibition of ENT1 causes an increase in extracellular adenosine, thereby resulting in a decrease in neuronal hyperexcitability and neurotransmission (Pandolfo et al. 2011). The study by Carrier et al. (2006) on the effect of CBD on the ENT1 revealed that CBD reduced the uptake of adenosine in microglial cells and RAW 264.7 macrophages in murine models. Carrier et al. showed that CBD enhances adenosine signalling by inhibiting the uptake of adenosine and providing a non-cannabinoid receptor mechanism through which it can reduce inflammation.

However, Carrier et al. (2006) reported that while binding to the ENT transporter, CBD can also activate the A_2A_ receptor. Moreover, blocking the uptake of adenosine by the microglial cells may impact the neurons which are neighbouring cells with the microglial cells in the brain parenchymal, thus causing a disruption in the activities and functions (such as cerebral blood circulation, learning) of these.

### Downregulation of TNF-α receptor

Tumour necrosis factor alpha (TNF-α) is a member of the 19 TNF superfamily that is secreted in the brain in response to pathogen invasions, such as bacterial and viral infections, as well as brain injuries. TNF-α is a cytokine used by the immune system for cell signalling, as it alerts other immune cells in the brain as part of an inflammatory response to infections or foreign bodies. TNF-α is also a major cytokine in neuroinflammation, which activates NF-KB, thereby causing glial activation and production of proinflammatory cytokines (Jung et al. 2019). Various studies report that CBD plays a role in downregulating TNF production.

In the study conducted by Mammana et al. (2019) on the efficacy of CBD and cannabigerol (CBG) in negating neuroinflammation using motoneuron-like cell line, it was observed that when used on its own, CBD reduced the concentration of TNF-α levels. Likewise, when used in combination with CBG, they both exerted anti-inflammatory effects on TNF-α. Aswad et al. (2022) investigated the effects of high concentrations of THC and CBD on cytokine production in immune cells both in vivo and in vitro; it was found that CBD caused significant reductions in pro-inflammatory cytokines in human T cells and neutrophils in vitro.

Also, the in vivo studies carried out by Aswad et al. (2022) using an inflamed mouse model revealed decrease in TNF-α and interleukin-1β (IL-1β) by a high concentration of CBD even though an increase in anti-inflammatory cytokine interleukin 10 (IL-10) was observed. According to Peyravian et al. (2020), CBD impedes important activators of the Janus kinase/signal transducer, transcription signalling pathway, and the nucleotide-binding oligomerization domain-like receptor signalling pathway which consequently reduces pro-inflammatory cytokine production. The downregulation of TNF-α indicates a reduction of brain damage. However, the downregulation of TNF-α by CBD does not guarantee the efficacy of CBD’s therapeutic ability as well as its safety and toxicity in chronic immune conditions. It is important to note that varying dosages of CBD influences its immunomodulatory properties, hence the need to understand the pharmacokinetics of CBD and the doses needed for modulation of the immune system.

### Restoration of BDNF expression

The brain-derived neurotrophic factor (BDNF) is one of the most active proteins, called neurotrophins, which help to stimulate and control the growth of new neurons from neural stem cells (neurogenesis) (Autry and Monteggia 2012). BDNF sustains nerve cells by aiding their growth, differentiation and maintenance (Soloey-Nilsen et al. 2022). BDNF also activates the tyrosine kinase receptors which recruit substrates such as phosphoinositide 3-kinases (PI3K) and the phospholipase c gamma (PLC-γ) in promoting neuron survival and neuroplasticity (Lin and Huang 2020). Being a crucial molecule in the development of neurons, BDNF performs vital roles in memory formation (spatial, long-term and emotional, particularly fear), which are linked to learning abilities; hence, BDNF is much implicated in psychiatric disorders (Autry and Monteggia 2012).

Decreased levels of BDNF are detrimental towards normal brain functioning as it plays a part in reducing the expression of synaptic proteins (which aids early neuronal development and regulation of neurotransmitter release), thereby leading to loss of synaptic connections and consequently the loss of neuronal ability to survive and adapt to environmental changes, and hence degeneration (Mariga et al. 2017). Reduction of BDNF also alters the ability of the nervous system to reorganize its structure and functions in response to intrinsic/extrinsic stimuli (Soloey-Nilsen et al. 2022). Apart from neuronal loss, reduced levels of BDNF have been linked with certain neurodegenerative and neuropsychiatric disorders such as Parkinson’s disease, Huntington’s disease, Alzheimer’s disease and mood disorders (Bathina and Das 2015). According to Lima Giacobbo et al. (2019), reduction in BDNF levels usually occurs as a result of long-term exposure to inflammation/neuroinflammation due to the negative effect of cytokines on BDNF-related signalling pathways.

CBD has been identified to be effective in the restoration of BDNF. From their studies, Mariga et al. (2017) and Sales et al. (2019) described the ability of CBD to stimulate and promote neurogenesis, cause drastic and lasting antidepressant-like effects through increased BDNF signalling, and formation of synapses in the prefrontal cortex, which is vital for the overall architecture of brain connections. These two studies also confirm that administration of CBD can increase the expression of the synaptic protein in the prefrontal cortex.

Winstone et al. (2022 doi.org/10.1089/can.2021.0025) administered 3 mg/kg of CBD to adolescent mice to evaluate the effect on BDNF, tropomyosin receptor kinase B (TrkB) and other synaptic markers. They observed that CBD significantly increased the expression of BDNF II, VI and IX, thereby causing growth stimulation as well as differentiation of synapses and neurons (neurogenesis). However, Winstone et al. 2022., reported a significant reduction in CB1 receptor protein in the ventral dentate gyrus (which regulates hormones and emotion), thus, the reduction in CB1 receptor may result in endocannabinoid dysregulation (Matias et al. 2006).

Moreover, differences were observed in the response of male and female mouse to CBD administration with the restoration BDNFII, VI and IX in females while CBD caused the restoration of only BDNF VI in males. Also, according to the report of Sales et al. (Sales et al. 2019), maintenance of a sustained elevated level of BDNF needs to be investigated.

### Restoration of brain 5-hydroxytryptamine levels

5-Hydroxytryptamine (5-HT), commonly referred to as serotonin, plays a significant role in modulating the release of several neurotransmitters such as epinephrine/norepinephrine, dopamine, and glutamate (Vaseghi et al. 2022). Prolonged periods of stress, faulty metabolism, and others factors that deplete the level of serotonin, have been linked with neurological disorders such as depression and anxiety (Kanova and Kohout 2021). In addition, production of proinflammatory cytokines such as IFN-ℽ and TNF-α results into tryptophan depletion by activating the quinolinic and kynurenine acid pathways, which makes tryptophan less available for the synthesis of serotonin (Richard 2020). In response to inflammation, following platelet activation, 5-HT is released for the regulation of all immune cells, mostly in their defence against infections, thus preventing excessive cytokine (chemokine) production and consequently cell damage and necrosis (Kanova and Kohout 2021). Serotonin does not only help in the defence of the body against infection/inflammation, but also hinders oxidative damage (Muñoz-Castañeda et al. 2006).

Linge et al. (2016) confirmed the efficacy of CBD in enhancing serotonin and glutamate levels in the ventromedial prefrontal cortex of mice in vivo*,* depending on the treatment duration (longer – two weeks – treatment with lower dose (10 mg/kg) yielded better results vs high dose (50 mg/kg) over one week). Following administration of CBD (100 mg/kg), Abame et al. (2021) observed a significant increase in 5-HT and noradrenaline levels in the hippocampus of C57BL 6 J mice. According to Jenny et al. 2010, CBD inhibits cytokine-induced tryptophan degeneration, and because tryptophan is a major compound in the synthesis of serotonin, increase in the bioavailability of tryptophan increases the levels of serotonin. However, chronic administration of CBD has been associated with other unwanted consequences such as anxiogenic effects as shown in the study of ElBatsh et al. (2012).

### Future of cannabidiol in treatment of neurological disorders/infections

Various anti-inflammatory drugs (such as dexamethasone) have been linked with adverse effects, ranging from moderate to severe, such as gastrointestinal side effects, allergic reactions and risks of cardiovascular conditions (van Bergen et al. 2022). However, CBD subdues inflammation with mild or negligible side effects, that do not pose a serious threat to the health of its users (Wheless et al. 2019). CBD has demonstrated safety and efficacy in the treatment of inflammation, making it a potentially more effective antidote with mild side effects that won't endanger or worsen users' health. Furthermore, CBD has further medicinal potential in addition to its anti-inflammatory qualities.

It has been shown that CBD is well tolerated, especially in its pure form, with less risk of cardiovascular and cerebrovascular effects (Harirforoosh et al. 2013). Based on its medicinal/therapeutic properties, CBD could proffer an unprecedented approach and unlock novel mechanisms for the treatment of brain-related disorders. CBD’s ability to suppress cytokines makes it a potentially useful agent in controlling neuroinflammation that may occur in brain infections, such as various forms of meningitis, thus mitigating the degree of neurological damage.

Owing to the campaign for the use of natural products in eradicating infections and their associated sequelae, as well as the curative effects of CBD, this phyto-cannabinoid would not only serve to assuage brain-related disorders but also serve as a natural panacea in inhibiting inflammation in illnesses *in lieu* of the traditional anti-inflammatory drugs. Considering the limitations of the non-steroidal anti-inflammatory drugs, it is necessary to regard the potential of CBD for use, on its own or as an adjunctive therapy, in the treatment of brain-related infections and its associated inflammation. The inherent polypharmaceutical properties of cannabis and CBD offer distinct advantages over the current single-target pharmaceutical model and portend to revolutionize neurological treatment into a new reality of effective interventional and even preventative treatment (Russo 2018).

However, the appropriate dosage of CBD that can penetrate the brain needs to be identified. This can be achieved through metabolomics (the study of the set of metabolites existing inside a biological system), which is a holistic approach to understanding various biological processes at the system level. This approach has not really been explored up to this point in cannabinoid research and will help to unveil the adequate concentrations of CBD and its metabolites that can penetrate the brain (especially when studied in animal models). The recent advancement of metabolomic techniques based on high-throughput analytical systems offers new ways to simultaneously detect, identify, and quantify a variety of chemical substances. In addition, there is need for more metabolomics studies on CBD to investigate the impact of this compound on neurological illnesses.

Applying metabolomics to the in vitro or in vivo investigation of CBD's effect(s) in different medical/health conditions, particularly by using animal models or cell cultures and clinical trials, will yield a wealth of data, uncovering and assisting in the identification of distinct chemical fingerprints that are the byproducts of physiological processes that take place in the cells. A direct functional assessment of the physiological condition of cells and tissues is provided by these chemical fingerprints that are left behind.

Metabolomics allows for the elucidation of the metabolite set that exists within a biological matrix, revealing complex and new phenotypic data. Therefore, the application of metabolomics will offer detailed information on the interaction and consequence of CBD in brain-related illnesses, proving the effectiveness of this compound in neurological conditions. Metabolomics will shed light and provide details on:

- The safety of CBD when used as a treatment for neurological conditions.
- The metabolic pathways influenced by CBD, and the phenotypic response.
- The influence of CBD on cytokine production and inflammatory biomarkers in brain-related disorders.

Therefore, any contraindications to using CBD for the treatment of neurological illnesses can be detected by studying how the drug reacts with the body.

Metabolite analysis provides access to a vast amount of untapped knowledge and studies in this regard can be used to highlight a variety of metabolic data. Therefore, applying metabolomics in CBD research for treating neurological illnesses will yield novel information at the metabolite (phenotypic) level.

## Conclusion

As reported in many studies, the reformative ability of CBD in neurological disorders is not only limited to the regulation of adenosine/oxidative stress but also the downregulation of TNF-α, restoration of BDNF mRNA expression and the recovery of serotonin levels, thus making it an important agent to consider in the production of immune suppressants and anti-inflammatory products in the future.

The ability of CBD to reduce oxidative stress indicates that in infections/neurological disorders, its administration would help in immune regulation by activating the adenosine receptors. Also, the rapid rise in cerebral adenosine, which disrupts the adenosine system and causes immune imbalances, can be managed by administration of CBD.

Despite all the remedial effects and potentials of CBD, the optimum dose of consumption has yet to be established. In addition, there are few studies that have evaluated the effect of CBD on chemokines and cytokine which occur from continuous systemic inflammations in response to certain forms of neurological infections, such as meningitis. Hence, additional studies with larger populations are recommended in this regard as this would not only provide sufficient and adequate findings but also assist in the application of experimental findings in clinical settings.

## Data Availability

Not applicable.

## Abbreviations

2-AG: 2-Arachidonoyl glycerol
5-HT: 5-Hydroxytryptamine
5-HT1A: 5-Hydroxy tryptophan 1A receptors
A_2A_: Adenosine A_2A_ receptors
AEA: N-Arachidonoylethanolamine
BDNF: Brain-derived neurotrophic factor
CB1: Cannabinoid receptor 1
CB2: Cannabinoid receptor 1
CBD: Cannabidiol
CBG: Cannabigerol
CB: Cannabinoid receptors
CYP2B6, CYP2C19, CYP2D6, CYP2J2: Cytochrome P450 enzymes
D-AMPH: D-amphetamine
ENT: Equilibrative nucleoside transporter
FAAH: Fatty acid amide hydrolase
FJB: Fluoro-JadeB
GPR: G protein-coupled receptors
HI: Hypoxic-ischaemic
iNOS: Inducible nitric oxide synthase
ICAM: Intracellular adhesion molecule 1
IFN-γ: Interferon-gamma
IL: Interleukin
IVHL: Ipsilateral hemisphere volume loss
JAK-STAT: Janus kinase-signal transducer and activator of transcription
LPS: Lipopolysaccharide
MAPK: Mitogen-activated protein kinase
MGAL: Monoacylglycerol acid lipase
NADPH: Nicotinamide adenine dinucleotide phosphate
NF-_K_B: Nuclear factor kappa B
NOX4: NADPH oxidase 4
NOX1: NADPH oxidase 1
Nrf2: Nuclear factor erythroid 2–related factor 2
PB: Polymyxin B
PI3K: Phosphoinositide 3-kinases
PLC-γ: Phospholipase c gamma
PPARℽ: Peroxisome proliferator-activated receptor gamma
RAW 264.7: Monocyte/macrophage cell line
RBD: Rapid eye movement sleep behaviour disorder
ROS: Reactive oxygen species
THC: Delta-9-tetrahydrocannabinol
TGFβ: Transforming growth factor beta
TNF-α: Tumor necrosis factor alpha
TrkB: Tropomyosin receptor kinase B
TRPV1: Transient receptor potential vanilloid
UGT1A7: UDP glucuronosyltransferase 1 family, polypeptide A7
UGT1A9: UDP glucuronosyltransferase 1 family, polypeptide A9
UGT2B7: UDP Glucuronosyltransferase Family 2 Member B7
UV: Ultraviolet
UVB: Ultraviolet B
XO: Xanthine oxidase
